# Correlation of Severity of Renal Colic With Clinical, Laboratory, and Radiological Parameters: An Emergency Department-Based Prospective Observational Study

**DOI:** 10.7759/cureus.31277

**Published:** 2022-11-08

**Authors:** Shrirang S Joshi, Nidhi Kaeley, Vempalli Nagasubramanyam, Pankaj Sharma, Alok Raj

**Affiliations:** 1 Emergency Medicine, All India Institute of Medical Sciences, Rishikesh, Rishikesh, IND; 2 Radiology, All India Institute of Medical Sciences, Rishikesh, Rishikesh, IND

**Keywords:** acute medical management of renal colic, renal stone disease, hydronephrosis, pyuria, hematuria, kidney calculi, emergency department, pain severity, acute renal colic

## Abstract

Introduction

In this study, we investigated the correlation of severity of renal colic with clinical parameters like pain characteristics, haematuria and pyuria, laboratory parameters such as inflammatory markers, and radiological parameters including site and size of stone and hydronephrosis.

Methods

The Visual Analogue Scale (VAS) determined the pain severity. Detailed history and clinicodemographic profiling of the patient was done, laboratory investigations were done, ultrasound and non-contrast computed tomography of kidney-ureter-bladder were done and all the parameters were duly noted and correlated with the pain severity.

Result

The mean age of the 183 patients was 43.96 ± 15.16 years, and 62.8% were male. The patients’ mean VAS score at presentation was 8.57 ± 1.08. The mean VAS score was found to be statistically higher in patients having a first episode of renal colic, solitary kidney, pyuria, raised creatinine, severe hydronephrosis, and stones located at the renal pelvis. In addition, higher VAS scores led to more surgical interventions.

Conclusion

The correlation of pain severity of renal colic with various parameters can aid in the development of quick diagnostic and therapeutic protocols for patients presenting to the emergency department with renal stone disease. This study shows that pain scores can correlate with various parameters and predict the outcome and complications in these patients.

## Introduction

Acute renal colic is a syndrome of flank pain with associated symptoms caused by nephrolithiasis [1]. It has a lifetime prevalence of around 5-15% and contributes around 31% to the entire acute abdomen presenting to the emergency department. Pain is usually acute and colicky in nature with or without radiation to the groin and thigh. Many patients report nausea, vomiting, hematuria, and pyuria as associated complaints [2,3]. Pathophysiologically, the degree of severity of pain correlates with the acuteness of obstruction than the degree of obstruction [4].

Management of renal colic includes laboratory investigations like complete blood count, urine analysis, renal function tests, and inflammatory biomarkers. Radiological investigations include ultrasound of kidney-ureter-bladder and non-contrast computed tomography of kidney-ureter-bladder. It also includes pain management and resuscitation followed by definitive treatment either surgical or conservative [5].

Pain management in renal colic possesses a challenge for emergency physicians. A number of studies have been done comparing the efficacy of various agents like non-steroidal anti-inflammatory drugs such as diclofenac and ketorolac, opioids, magnesium sulfate, lidocaine, nitrates, anti-muscarinic agents such as hyoscine butylbromide, etc. [6]. However, studies related to the severity of pain are limited.

In our search of the literature, we found few studies correlating various parameters with pain severity. A study by Shih et al. reported the correlation of epidemiological and clinical parameters with pain severity. Other studies by Lallas et al., Dorfman et al., and Sasmaz and Kirpat reported a significant correlation of pain severity with hematuria and pyuria [7-10]. Gourlay et al. and Splinter et al. reported that there was no significant correlation of pain with stone size, location, and patient outcomes. On the other hand, Portis et al. in a study found that pain score was an independent predictor of surgical outcome [11-13]. However, a study by Sasmaz and Kirpat found a significant correlation of pain severity with hydronephrosis and biomarkers like C-reactive protein and Neutrophil-Lymphocyte ratio [10]. No such study was found conducted in the Indian setting.

In this prospective observational study, we aim to determine the correlation of severity of pain of renal colic with clinical, radiological, and laboratory parameters to overcome the shortcomings of previous studies and fill in the literature lacunae. This research will aid in a better understanding and adequate control of pain and management of patients with renal calculi. This will help by not only decreasing their emergency department and hospital stay but also minimizing the complications, resulting in improved mortality and morbidity.

## Materials and methods

Study design and setting

This prospective observational study was performed at the Emergency Medicine Department, All India Institute of Medical Science, Rishikesh, over a period of 18 months from October 2020 to March 2022.

Selection of participants

Participants were selected on the basis of inclusion and exclusion criteria. Patients included in the study were adults (more than 18 years) with unilateral flank pain attributed to renal colic with concomitant radiological evidence of renal stone disease who volunteered to participate in the study. Patients with concomitant abdominal pathology which mimics renal colic, pregnant females and patients with age less than 18 years, patients with bilateral or diffuse pain, and patients whose pain score cannot be assessed were excluded from the study.

Sample size estimation

Sample size was calculated based on Cohen’s convention for sample size estimation. Medium effect size=0.25 was assumed at 80% power with a level of significance at 0.05. The estimated sample size came out to be 180.

Method and measurement

**Figure 1:**
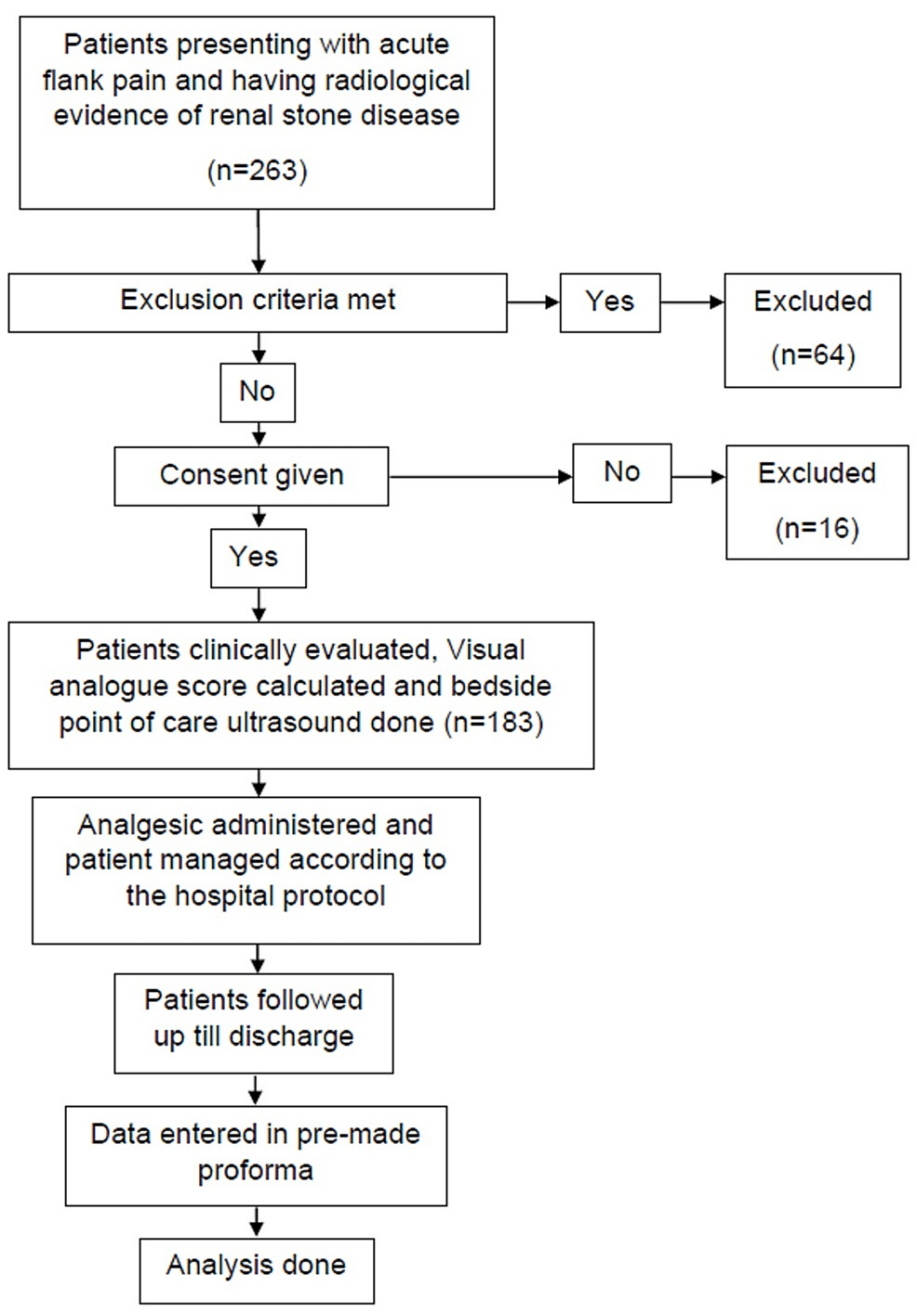
Study flowchart .

Detailed demographic profile of patients presenting to the emergency department with unilateral flank pain attributing to renal colic was noted by the principal investigator who was an emergency medicine resident. A thorough history and physical examination were done. Point-of-care ultrasound was done by the principal investigator after completing the academic training in point-of-care ultrasound and he had performed at least 25 scans under the supervision of a radiologist [14,15]. The patient was given appropriate analgesia after noting the severity of pain which was measured in terms of the visual analog scale (VAS). Relevant laboratory and radiological investigations were sent and for each patient, a urology consultation was sought. The patient was enrolled in the study after informed consent (Figure 1).

The following were the criteria of clinical parameters:

1. Severity of pain: Visual Analogue Scale (VAS) 0 to 10 (0=no pain, 1-3=mild, 4-6=moderate, 7-10=severe) [16]

2. Hematuria: more than or equal to 5 red blood cells per high power field [9]

3. Pyuria: more than or equal to 10 pus cells per high power field [9]

4. Hydronephrosis: Distension of renal pelvis and calyces due to urinary outflow obstruction. Grades - mild, moderate and severe according to the Society of Fetal Urology [17].

Outcomes

The primary objective was to determine the correlation of severity of renal colic measured in terms of VAS score and compare it with various parameters.

Ethical approval

Ethical approval for the study was obtained from the Institute Ethics Committee, All India Institute of Medical Sciences, Rishikesh (AIIMS/IEC/20/664 Date:03/10/2020).

Statistical analysis

The data was entered in an Excel sheet and analyzed with SPSS software version 23 (IBM Corp., Armonk, NY). Various parameters were correlated with the VAS score. A p-value < 0.05 (two-tailed) was considered significant.

## Results

A total of 183 patients were enrolled in the study after considering the inclusion and exclusion criteria, out of which 115 (62.8%) were males. Obstructive uropathy was diagnosed in 166 (90.7%) patients. The mean number of renal colic episodes in past was 1.01 ± 0.96. The pain was predominantly colicky and burning micturition (n=22; 12%) was the most common associated symptom after haematuria (n=61; 33.3%) and pyuria (n=56; 30.6%) (Table 1).

**Table 1:** Demographic and clinical profile of patients with unilateral renal colic (n=183)

Variable	Mean ± Standard Deviation (SD) || Frequency (%)
Age (Years)	43.96 ± 15.16
Gender	
Male	115 (62.8)
Female	68 (37.2)
Final Diagnosis	
Obstructive Uropathy	166 (90.7)
Non-Obstructive Uropathy	17 (9.3)
Number of Episodes in Past	1.01 ± 0.96
Pain characteristics	
Side of pain	
Right	99 (54.1)
Left	84 (45.9)
Character	
Colicky	174 (95.1)
Dull aching	9 (4.9)
Radiation to groin and genitalia	
Present	141 (77.0)
Absent	42 (23.0)
Associated symptoms	
Burning micturition	22 (12.0)
Fever	16 (8.7)
Vomiting	12 (6.6)
Anuria	9 (4.9)
Oliguria	6 (3.3)
None	118 (64.5)
Haematuria	
Present	61 (33.3)
Absent	122 (66.7)
Pyuria	
Present	56 (30.6)
Absent	127 (69.4)
Heart rate (beats per minute)	101.13 ± 17.43
Blood pressure (mm of Hg)	
Systolic blood pressure (mm of Hg)	133.99 ± 17.38
Diastolic blood pressure (mm of Hg)	82.99 ± 9.65
Respiratory rate (per minute)	24.50 ± 5.23

Among clinical parameters, a significant correlation was found with the final diagnosis of the patient. The mean visual analogue scale (VAS) score in the obstructive uropathy group was found to be significantly higher than in the non-obstructive uropathy group (W = 879.500, p = 0.006). Also, there was a statistically significant negative correlation between the number of renal colic episodes in the past and VAS (rho = -0.27, p = <0.001). For every one-unit increase in the number of episodes in past, the VAS decreased by 0.18 units, signifying that the severity of pain was highest in the first episode and subsequently decreased with later episodes. Out of all the patients investigated, 29 (15.8%) had hypertension, which was the most common comorbidity followed by diabetes (n=168.7). Among the co-morbidities, the median VAS in the solitary kidney group was found to be significantly higher than in the other patients (W = 502.500, p = 0.006). We also found a significant correlation between the severity of pain and pyuria (W = 4304.500, p = 0.015). No correlation was found between the severity of pain and haematuria (p=0.099) (Table 2).

**Table 2:** Correlation of VAS score with various clinical and demographic parameters in patients with unilateral renal colic (n=183) Significant at p<0.05, 1: Spearman Correlation, 2: Wilcoxon-Mann-Whitney U Test VAS: Visual analogue scale

Parameters	Mean ± SD || Frequency (%)	VAS (mean ± SD)	p-value	Correlation coefficient
Age (Years)	43.96 ± 15.16		0.231^1^	rho = 0.09
Gender			0.108^2^	W = 3392.000
Male	115 (62.8)	8.46 ± 1.15		
Female	68 (37.2)	8.75 ± 0.94		
Final Diagnosis			0.006^2^	W = 879.500
Obstructive Uropathy	166 (90.7)	8.50 ± 1.08		
Non-Obstructive Uropathy	17 (9.3)	9.24 ± 0.83		
Number of Episodes in Past	1.01 ± 0.96		<0.001^1^	rho = -0.27
Side of Pain			0.283^2^	W = 3392.000
Right	99 (54.1)	8.47 ± 1.17		
Left	84 (45.9)	8.68 ± 0.96		
Co-Morbidities:				
None	113 (61.7)	8.62 ± 1.01	0.626^2^	W = 4113.000
Hypertension	40 (21.8)	8.40 ± 1.37	0.734^2^	W = 2766.000
Diabetes	27 (14.7)	8.89 ± 1.09	0.070^2^	W = 2534.000
Chronic Kidney Disease	11 (6.0)	8.73 ± 0.47	0.962^2^	W = 954.000
Solitary Kidney	3 (1.6)	10.00 ± 0.00	0.006^2^	W = 502.500
Haematuria			0.099^2^	W = 4239.500
Present	61 (33.3)	8.75 ± 0.98		
Absent	122 (66.7)	8.48 ± 1.12		
Pyuria			0.015^2^	W = 4304.500
Present	56 (30.6)	8.84 ± 1.02		
Absent	127 (69.4)	8.45 ± 1.09		

VAS score was significantly correlated with hematological parameters such as neutrophils (p=0.015; rho=-0.18), lymphocytes (p=0.045; rho=0.15) and monocytes (p=0.003; rho=0.22). There was a weak negative correlation found with neutrophil fraction and a positive correlation was found with lymphocytes and monocytes. Also, there was a weak positive correlation between creatinine and VAS (rho = 0.23, p = 0.002). For every 1 unit increase in creatinine, the VAS increased by 0.30 units (Table 3).

**Table 3:** Correlation of VAS score with laboratory parameters in patients with unilateral renal colic (n=183) Significant at p<0.05 VAS: Visual analogue scale

Parameter	p-value	Spearman correlation coefficient (rho)
Total leukocyte count	0.405	0.06
Differential leukocyte count		
Neutrophils	0.015	-0.18
Lymphocytes	0.045	0.15
Monocytes	0.003	0.22
Eosinophils	0.066	0.14
Basophils	0.195	0.1
C-reactive protein	0.819	-0.02
Creatinine	0.002	0.23
Urea	0.491	-0.05

Out of all, 144 (78.7%) had varying degrees of hydronephrosis on the side of pain on bedside point-of-care ultrasound evaluation. Maximum number of patients were found to have moderate hydronephrosis (n=79; 43.2%). Stone was most commonly found in the upper ureter (n=50, 27.3%). Among radiological parameters, the mean VAS score was higher in the patients with severe hydronephrosis when compared with patients having mild, moderate, or no hydronephrosis on bedside point-of-care ultrasound (POCUS). Although the difference was minimal, it yielded a significant correlation (p=0.029; χ2=9.058). A similar correlation was also obtained with the VAS score when evaluated against hydronephrosis detected by non-contrast computed tomography of kidney-ureter-bladder (NCCT KUB) (p=0.045; χ2=8.036). Although, no trend of increasing severity of pain was observed with increasing hydronephrosis. Also, a significant correlation was found between the site of stone and VAS score (χ2 = 11.906, p = 0.036), with the median VAS being highest when the stone was located in the renal pelvis when compared to stones located at other sites (Table 4).

**Table 4:** Correlation of VAS score with radiological parameters in patients with unilateral renal colic (n=183) Significant at p<0.05, 1: Spearman Correlation, 2: Kruskal-Wallis Test VAS: Visual analogue scale

Parameter	Frequency (%) || Mean ± SD	VAS score (mean ± SD)	p-value	Correlation coefficient
Degree of Hydronephrosis on side of pain on POCUS			0.029^2^	χ2 = 9.058
No Hydronephrosis	39 (21.3)	8.77 ± 1.22		
Mild	35 (19.1)	8.71 ± 0.99		
Moderate	79 (43.2)	8.30 ± 1.12		
Severe	30 (16.4)	8.83 ± 0.70		
Degree of Hydronephrosis on side of pain on NCCT KUB			0.045^2^	χ2 = 8.036
No Hydronephrosis	39 (21.3)	8.77 ± 1.22		
Mild	24 (13.1)	8.71 ± 1.16		
Moderate	91 (49.7)	8.37 ± 1.06		
Severe	29 (15.8)	8.79 ± 0.77		
Size of Largest Stone (mm)	11.79 ± 6.43		0.336^1^	rho = -0.07
Site of Stone on side of pain			0.036^2^	χ2 = 11.906
Pelvis	14 (7.7)	9.14 ± 1.10		
Pelvi-ureteric junction	33 (18.0)	8.85 ± 1.00		
Upper Ureter	50 (27.3)	8.28 ± 1.14		
Mid Ureter	13 (7.1)	8.77 ± 0.44		
Lower Ureter	34 (18.6)	8.50 ± 1.05		
Vesico-ureteric junction	39 (21.3)	8.49 ± 1.14		

The mean VAS score in patients who underwent surgical intervention was found to be higher than in patients who were managed conservatively. Hence, it was found that the severity of pain significantly correlated with the need for surgical intervention (p=0.001; W=4791.000) (Table 5).

**Table 5:** Correlation of VAS score with outcomes in patients with unilateral renal colic presenting to the emergency department (n=183) Significant at p<0.05, 1: Spearman Correlation, 2: Wilcoxon-Mann-Whitney U Test VAS: Visual analogue scale

Outcomes	Mean ± SD || Frequency (%)	VAS score (mean ± SD)	p-value	Correlation coefficient
Nature of Disposition from Emergency Department			0.071^2^	
Discharged	68 (37.2)	8.35 ± 1.18		
Admitted	115 (62.8)	8.70 ± 1.00		
Length of Stay in Emergency Department (Hours)	4.64 ± 1.69		0.825^1^	rho = - 0.02
Surgical Intervention			0.001^2^	W = 4791.000
Done	62 (33.9)	8.90 ± 1.05		
Not done	121 (66.1)	8.40 ± 1.06		

## Discussion

Our study was a prospective observational study to determine the correlation between pain severity of renal colic and various clinical, laboratory and radiological parameters. It was carried out in a tertiary care centre in north India, at the All India Institute of Medical Sciences Rishikesh.

Our study found that among clinical parameters, the severity of pain did not correlate with age, gender, pain characteristics or associated symptoms. However, pyuria significantly correlated with the severity of pain (p=0.015). Similar results were also obtained by Sasmaz and Kirpat (p=0.003) [10]. Higher pain scores in patients with pyuria in renal colic could be attributed to the presence of concomitant urinary tract infection. Renal stones by causing stasis of urine can form a nidus for infection. No such correlation was found with hematuria (p=0.099). It was contrary to Lallas et al. who found that pain scores were significantly greater in patients who presented with hematuria (p=0.0147) [8]. Sasmaz and Kirpat found that the mean VAS score was higher in patients having hematuria when compared to those who didn’t (p=<0.001) [10]. Abrahamian et al. reported about 8% of patients with acute renal colic had urinary tract infection. However, pyuria had moderate accuracy in detecting urinary tract infections [18].

The severity of pain was significantly higher in the non-obstructive uropathy group than in the obstructive uropathy group (mean VAS score in non-obstructive uropathy group = 9.24 + 0.83; W = 879.500; p = 0.006). Although small non-obstructing stones with a size of < 5 mm (Golan et al.) or < 4 mm (Jura et al.) are considered to be painless, there are few patients who present with ‘the small stone syndrome’. They have significant pain and are considered to be having a small stone without any radiological obstructive feature. Studies by Jura et al. and Golan et al. reported that these patients possess a challenge for the managing physician [19,20].

In our study, the mean number of previous episodes of renal colic was 1.01 ± 0.96 ranging from 0 to 4. The pain severity decreased with subsequent episodes (p=<0.001 correlation coefficient = - 0.27). This could be attributed to the development of pain tolerance. No previous study has reported such an association in the past.

When compared with other co-morbidities, patients with solitary kidney experienced more severe pain. However, only three patients were enrolled in this group (n=3; p=0.006). No similar study was found regarding the correlation of severity of pain in patients with solitary kidney.

Among laboratory parameters studied, VAS score has a weak positive correlation with lymphocyte count (rho = 0.15, p = 0.045), and monocyte count (rho = 0.22, p = 0.003). It also showed a negative correlation with neutrophils (rho = -0.18, p = 0.015). We did not find any correlation of severity of pain with total leukocyte count. Sasmaz and Kirpat in an observational study found a very weak positive correlation with total leukocyte count (p<0.001, Pearson correlation coefficient=0.218) and neutrophil-lymphocyte ratio (p<0.001, Pearson correlation coefficient=0.220) [10].

The mean C-reactive protein (CRP) was 3.71 ± 1.42. Alleemudder et al. found the mean CRP to be 15.9 (1-192) which was significantly higher than our results [21]. We did not find any significant correlation between CRP and the severity of renal colic (p=0.819). Contrarily, Sasmaz and Kirpat found a significant correlation between the severity of pain and CRP (p=<0.001; r=0.276) [10]. Higher CRP levels could be attributed to the local inflammation of the urinary tract or the presence of urosepsis in a patient with recurrent stone disease.

There was a weak positive correlation between serum creatinine and VAS score (rho = 0.23; p = 0.002) in our study.

Among radiological parameters, 78.7% of patients had varied degrees of hydronephrosis on imaging. On grading, it was found that the severity of pain was significantly higher in patients having severe hydronephrosis (χ2 = 9.058, p = 0.029). The severity of renal colic is correlated with the acuteness of obstruction rather than the degree of obstruction. Pathophysiologically, higher built up of upstream pressures resulting in more distension of renal capsule and ureteral lumen can cause more severe pain, particularly in an acute setting. Sasmaz and Kirpat found that the mean VAS score of the patients with hydronephrosis was statistically higher than those without hydronephrosis (p < 0.001) [10]. However, Splinter et al. did not find any significant correlation between arrival pain scores and the degree of hydronephrosis (b = 0.016; 95% CI: -0.053, 0.022, p = 0.418) [12].

The mean stone size was found to be 11.79 ± 6.43 mm. There was no significant correlation between the size of the stone and the severity of pain (p=0.336). A similar study done by Sasmaz and Kirpat found no correlation between stone size and VAS pain score (r = 0.079, p = 0.123) [10]. In a study done by Gourlay et al., they found a weak negative association (adjusted OR=0.96) between pain severity and stone width indicating that smaller stones were more symptomatic [11]. Contrarily, the study by Splinter et al. which correlate arrival pain scores with stone size also found that smaller stones caused more pain when compared to larger stones (b=−0.0004; 95% CI: -0.0015; 0.0008) [12]. Similar results were also obtained by Shih et al. (p=0.025) [7].

In our study, stones located in the renal pelvis were found to cause more severe pain when compared to stones at other locations (χ2 = 11.906, p = 0.036). In a similar study by Lallas et al., 70.6% of ureteral and renal pelvic stones resulted in the presence of one or more stone-related symptoms compared with 16.9% of all caliceal stones (p = 0.0001). Also, it was observed that proximal ureteral stones caused the highest rate of symptoms (100%) [8]. Splinter et al. in a similar study correlating arrival pain scores in renal colic with the location of stone did not find any significant correlation (b = 0.0045; 95% CI: -0.020, 0.029) [12].

In our study, 62 (33.9%) patients required surgical intervention. The most common intervention was DJ stenting (42; 67.8%), followed by percutaneous nephrolithotomy (12; 19.4%) and extracorporeal shock-wave lithotripsy (8; 12.8%). There was a significant correlation between the severity of pain and the need for surgical intervention in our study. Patients with higher VAS score were more likely to have a surgical intervention (P=0.001). This could be attributed to the more acuteness of obstruction to urinary flow that can be surgically relieved to ease the pain. Contrarily, a study by Gourlay et al. did not find any association between the severity of pain and the need for hospitalisation or rescue intervention within 60 days [11]. A study by Portis et al. found that pain severity was an independent predictor of the need for surgical intervention [13]. However, a study by Innes et al. found that early intervention resulted in higher rates of emergency department revisits and rehospitalization [22].

Thus, this study implies that these correlations between pain severity and various parameters can help us make prompt clinical decisions for patients with acute renal colic rather than waiting for laboratory or imaging results.

However, the limitations of this study include:

1. Altered patient inflow due to the COVID pandemic.

2. Hemodynamically unstable patients were excluded from the study.

3. Anthropometric parameters and nutrition parameters were not included as it was not feasible in the emergency department.

4. Procalcitonin was not included in biomarkers as it was not feasible to investigate in all the patients. Correlation with these can be evaluated in further studies.

## Conclusions

The correlation of pain severity of renal colic with various parameters can aid in the development of quick diagnostic and therapeutic protocols for patients presenting to the emergency department with renal stone disease. This study will not only help in better understanding of patients with renal colic but also the management of varied complications of renal stone disease. As the severity of pain increased with rising serum creatinine and pyuria and is positively correlated with hydronephrosis and stone location, the emergency physician can be more prompt in ruling out these complications in patients with severe renal colic.

## Appendices

Patient proforma

Patient proforma can be found online at the following google form link:

https://docs.google.com/forms/d/e/1FAIpQLScMYcYF5BhB5tJ0iR8viuaIooKDpwzGoYdzg9SU89d4XhXZEg/viewform?usp=sf_link

Participant information sheet

**Table 6:** Participant information sheet

Participant Information Sheet	
You/your patient is being invited to participate in a research study. Before you take part in this research study, we wish to explain the study to you and give you the chance to ask questions. Please read carefully the information provided here. If you agree to participate, please sign the informed consent form.	
STUDY TITLE	Correlation of severity of renal colic with clinical laboratory and radiological parameters - an emergency department-based prospective observational study
INVESTIGATOR	Dr. Shrirang Shriram Joshi, Post graduate student, Department of Emergency Medicine, AIIMS Rishikesh.
GUIDE	Dr. Nidhi Kaeley, Associate Professor, Department of Emergency Medicine, AIIMS Rishikesh
METHOD	This is a hospital-based prospective observational study. If the patient satisfies the inclusion and exclusion criteria, he/she will be enrolled for the study. General Physical Examination which will include Vitals- temperature, examination of blood pressure, Pulse rate, respiratory rate and oxygen saturation will be done. This will be followed by a systemic examination and bedside ultrasound (point-of-care ultrasound). Lab Investigations namely Total leukocyte count, Differential leukocyte count, Urine routine microscopy for hematuria and pyuria and CRP will be done as per treating physician. Radiological investigations will be done as per treating physician’s advice to assess the size and location of stone and the degree of hydronephrosis, which will include Ultrasound KUB and NCCT KUB. All patients will be treated as per urologist’s advice.
BENEFITS OF THE STUDY	By obtaining and analysing the data, we can determine whether there is any association of severity of renal colic with various clinical, laboratory and radiological parameters.
WITHDRAWAL FROM STUDY	You have the right to withdraw from study at any time during the course of the study without any prejudice to your or your family`s right to undergo future treatment at AIIMS Rishikesh. Your decision not to participate in the research study will not affect the medical care in this institution.
SUBJECT RIGHTS	It is your right to decide whether or not to take part in this study. You are free to ask if you have any queries or concerns.
CONFIDENTIALITY OF STUDY AND MEDICAL RECORDS	Records of your study participation will be kept confidential. Any publication of data will not identify you by name. By signing the consent form you authorize the sharing of your study-related medical records to the regulatory authorities and the Institutional Ethical Committee.
WHOM TO CONTACT IF YOU HAVE QUESTIONS	Dr. Shrirang Shriram Joshi, Junior Resident, Emergency Medicine AIIMS Rishikesh, 9811540152; Dr. Nidhi Kaeley, Associate Professor, Emergency Medicine, AIIMS Rishikesh

Consent form in English

Participant identification number for the study:

STUDY TITLE: “Correlation of renal colic with various clinical, laboratory and radiological parameters: an emergency department-based prospective observational study”

INVESTIGATOR: Dr. Shrirang Shriram Joshi, PG student.

I ______________ (consent giver) _________ (relation to patient/self) of _______________ (name of patient) a resident of _______________ do hereby declare that I give informed consent to participate in this study and do agree to comply with the study design for academic and scientific research. The contents of the information sheet that was provided have been read carefully by me / explained in detail to me, in a language that I comprehend, & I have fully understood the contents. I confirm that I have had the opportunity to ask questions. The nature and purpose of the study and its potential risks / benefits and expected duration of the study, and other relevant details of the study have been explained to me in detail, I understand that my / my patient’s participation is voluntary and that I am free to withdraw at any time, without giving any reason, without my / my patient’s medical care or legal right being affected. I have understood that the sponsors of the study, the people working on behalf of the sponsor, the Ethics Committee and the regulatory authorities do not need my permission to see my health records in reference to the current study or further study; regardless of if I have withdrawn my name from this study. However, I understand that my identity will not be given in any third party or published medium. I agree that there is no restriction on my behalf for the use of the scientific purpose (s) of any data or results obtained from this study. I have been told that the tests/investigations done are not harmful. I agree to take part in the above study.

______________________ Name of the participant/ relative Signature/Left thumb impression of (participant /relative) Date: - Place: -

We have witnessed that the patient signed the above form in the presence of his/her free will after fully having understood its contents.

Witness 1

_____________ Signature:

Name:

Address:

Witness 2 _____________

Signature

Name:

Address:
